# The Serum Pepsinogen Test as a Predictor of Kazakh Gastric Cancer

**DOI:** 10.1038/srep43536

**Published:** 2017-03-02

**Authors:** Wei Juan Cai, Liang Yin, Qiang Kang, Zu Chen Zeng, Shao Liang Wang, Jiang Cheng

**Affiliations:** 1 Department of Clinical Laboratory, the First Affiliated Hospital, Shihezi University School of Medicine, Shihezi, Xinjiang 832002, P.R. China; 2 Department of Endocrinology and Metabolism, Tongji Hospital, Tongji Medical College, Huazhong University of Science and Technology, Wuhan, Hubei 43000, P.R. China

## Abstract

Gastric cancer (GC) is one of the most common malignant tumors and the Kazakh population in Xinjiang has been reported to be one of the highest incidence of GC in the world. Serum pepsinogen (PG) test provides a valuable method for detecting GC, but little study about the role of PG in Kazakh GC. Therefore, we aimed to investigate the role of PG in Kazakh GC and to elucidate the usefulness of the serum PG test method. The serum PG concentration were measured using the flow fluorescence assay and ELISA methods in patients with superficial gastritis, atrophic gastritis and GC. The most suitable cut off point was a PG I concentration ≤64 ng/ml and PG I/II ratio (PGR) ≤4.5. Using this cut off point, the sensitivity and specificity of pepsinogen screening for Kazakh GC were 80.5% and 89.8%, respectively. The area under the curve (AUC) of the PGR for GC diagnosis was 0.949, which was significantly higher than that of combined tumor markers. Moreover, PGR in Kazakh early GC was statistically significantly lower than in SG and AG. These findings suggest that serum PG test can serve as a noninvasive biomarker for the diagnosis of Kazakh GC.

Gastric cancer (GC) is one of the most common malignant tumors in the world. It has been estimated that a total of 989,600 new GC cases and 738,000 deaths occurred in 2008^1^. The incidence rate varies in different physiographical regions, nations and races. China has a high incidence of GC with a high mortality rate for these patients^2^. In particular, GC is the leading cause of cancer death, with a mortality rate of 31.6/100,000 in the Kazakh ethnic group residing in northwest Xinjiang Province of China, which is higher than any other ethnic group in China^3^. Unfortunately, tumor biomarkers such as CEA and CA19-9 that are currently utilized for the detection of GC in clinical practice are not specific and sensitive enough^4^. Therefore, novel diagnostic biomarkers and new methods are urgently required for mass surveys of early events of Kazakh GC.

Pepsinogens (PG) originating from gastric mucosa can be classified into two immunochemically distinct groups: pepsinogen I (PG I) and PG II^5^, which are mostly secreted into the gastric lumen and nearly 1% of them are leaked into the blood circulation. Serum PG levels reflect the morphological and functional status of gastric mucosa^6^,^7^. serum PG tests served as a useful marker for the prevalence of GC in a cross sectional setting^8^. At present, the commonly used methods for PG detection are radioimmunoassay (RIA), ELISA, chemiluminescence (CLIA), time-resolved fluorescence immunoassay (TrFIA) and latex-enhanced immunoturbidimetric assay^9^,^10^. Streaming fluorescence technique is a kind of high throughput detection technology which based on fluorescence coded microspheres and streaming technology. It has the advantages of high flux, high matching reagent, wide applications and good repeatability, and can not only detect the protein but also can detect the nucleic acid.

Although the aberrant serum PG concentration has been reported in GC, serum PG concentration in Kazakh GC is unclear. Thus, the aim of the present study was to detect serum PG concentration in Kazakh GC using flow fluorescence technology. Furthermore, we compared the accuracy between the PG I/II quantitative assay kit (flow fluorescence method) and ELISA kit method and elucidated the usefulness of the serum PG test method. Through this process, we were able to establish a sensitive and efficient screening method for the detection of Kazakh GC using serum PG concentration.

## Results

### Clinical characteristics

Totally, 464 subjects were recruited sequentially from the Yili Friendship Hospital. Study groups included normal control (NC, n = 160, mean age 42.5 ± 12.5 years), superficial gastritis (SG, n = 102, mean age 30.6 ± 10.5 years), atrophic gastritis (AG, n = 96, mean age 44.7 ± 7.4 years), gastric cancer (n = 106, mean age 53.3 ± 13.8 years). Among them, the patients of gastric cancer were categorized into two groups, early gastric cancer (EGC) (n = 64, mean age 52.7 ± 12.6 years), advanced gastric cancer (AGC) (n = 42, mean age 57.7 ± 15.3 years). Table 1 shows the characteristics of the GC groups. There were 52 (49.1%) of intestinal and 50 (47.2%) of diffuse type cancers according to Lauren classification. Meanwhile, the percentage of smoking and alcohol are shown in Table 1. About 72 (67.9%) patients whose GC was identified by investigational endoscopy without any symptom. Only 34 (32.1%) patients complained of a burning sensation or constriction in the epigastrium.

### The limit of detection

The zero calibration kit (CAL1) which as the blank sample, parallel determination of 20 blank samples to obtain the corresponding value of the fluorescence signal, calculate their mean ± SD. The corresponding concentration of fluorescence signal value as the lowest detection limit of each index. PG I, PG II index minimum detection limits were 0.87 ng/ml and 0.46 ng/ml.

### The linear range of the verification

**Figure 1:**
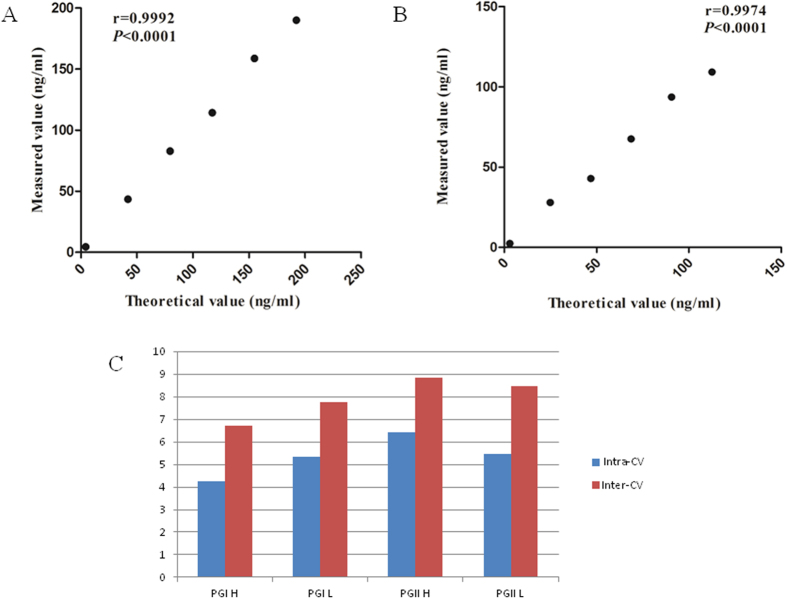
(**A**) Linear regression curves of relative PG I levels and theoretical value. (95% Confidence Intervals for the slope: 0.9927 to 0.9999, *P* < 0.0001, r = 0.9992). (**B**) Linear regression curves of relative PG II levels and theoretical value. (95% Confidence Intervals for the slope: 0.9749 to 0.9997, *P* < 0.0001, r = 0.9974). (**C**) Precision evaluation data of different concentration levels of PG I and PG II.

Reference EP6-A file for linear range verification. The linear ranges of PG I and PG II provided by the kit were 1 ng/ml~180 ng/ml and 1 ng/ml~100 ng/ml. Collection of specimens from the clinical which approach detecting upper and lower limit of detection. PG I and PG II high values were 192.10 ng/ml and 112.57 ng/ml, and the low values were 4.20 ng/ml and 2.97 ng/ml, respectively. Low value and high value specimens in accordance with 1:0, 1:4, 2:3, 3:2, 4:1 and 0:1 volume ratio of mixed into 6 concentration levels of the sample. Samples for each level repeated the test twice and calculated the average. After that, we performed regression analysis of the measured average value and the theoretical value. The linear range of verification results shown in Table 2, the r values of PG I and PG II were 0.9992 and 0.9974, respectively (Seen in Fig. 1A and B). The results showed that PG I, PG II detection had good linearity within the linear range of the kit.

### Serum PG precision measurement

Reference to the US Committee for Clinical Laboratory Standards (NCCLS) EP5-A2 document standards for testing and data processing. Low and high value specimens of PG I and PG II were detected by flow fluorescence method, and did two batches of test every day. Each batch of test of double measurement of high and low value samples, and did a total of 20d which finally got 80 test results of PG I and PG II, respectively. Then, the intra- and inter-assay coefficient of variation (CV) were seen in Fig. 1C.

### Recovery experiment

Select routine test samples, the concentration of PG I was 4.98 ng/ml, and PG II was 3.43 ng/ml. Volume was divided into the same three parts. Two parts were recovered analysis samples which were different the added concentrations, and calculated the analyte concentration of the added. Another part which was base sample. Recovery of data shown in Table 2.

### Anti-jamming capability assessment

In order to recover the anti-interference ability of the reagent box, serum samples with different concentrations of PG I and PG II were added triglyceride, bilirubin and hemoglobin (Hb). The results shown that PG I and PG II recoveries were between 85% to 115% in added 200 mg/mL triglyceride, 10 mg/ml of bilirubin and Hb respectively. Its non-interference kit, shown in Table 3.

### Specificity evaluation

PG I pure antigen PG II which was from Sigma Diagnostics Company. Diluted to 400 ng/ml which as a high value sample of PG. In addition, the use of PG II pure antigen without PG I which was from Meridian Life Science Company. Diluted to 150 ng/ml, as a high-value PG II samples. Used the kit for testing, the results were showed that the concentration of PG II was 0.27 ng/ml and PG I > 180 ng/ml in the PG I high value samples. The detection concentration of PG I was 0.86 ng/ml and PG II > 100 ng/ml in the high value samples of PG II.

### Hook effect evaluation

Take PG I and PG II pure antigen which original concentration were 0.35 mg/ml and 0.1 mg/ml. First of antigen after 10-fold dilution, and then the diluted sample for continuous double dilution which can get twelve gradient concentrations of the samples. In the measured concentration and the theoretical concentration were not proportional to the increase and greater than the upper limit of the kit the maximum theoretical concentration which as kit appears concentration Hook effect. The results shown that PG I and PG II did not appear Hook effect at 16301 ng/ml and 7330 ng/ml, respectively.

### Methodology comparison

Levels of serum PG I and PG II in 143 patients which were detected by flow fluorescence method (Y). And the correlation regression analysis was carried out with the results of ELISA detection (X). Then the regression equation was established. By calculation, linear regression equation between two methods of PG I which was Y = 0.911X-22.635 (r = 0.966, *P* < 0.05). And linear regression equation of PG II was Y = 0.892X-0.548 (r = 0.980, *P* < 0.05). The results shown that the two methods of PG I and PG II had a good correlation. System error was small, and there was a good comparability.

### Reference intervals established

160 serum samples from healthy subjects (NC) which were collected from the Yili Friendship Hospital. Among them, 78 were male and 82 were female. <20 years (13 cases), 20~39 years (63 cases), 40~60 years (57 cases), >60 years (27 cases). The samples were tested according to the reagent kit and the detection results were collected. And PG I and PG I/II ratio (PGR) data was carried out normality test. The results shown that both the data was not normally distributed, therefore, used the percentile method to establish the reference range of PG I and PGR. Fifth percentile (P5) of PG I was 72.77 ng/ml and for first percentile (P1) was 32.53 ng/ml. P5 of PGR was 4.16 and for P1 was 3.47. Used P5 as the lower limit of the reference interval of PG I and PGR.

### Test results each experimental group

**Figure 2:**
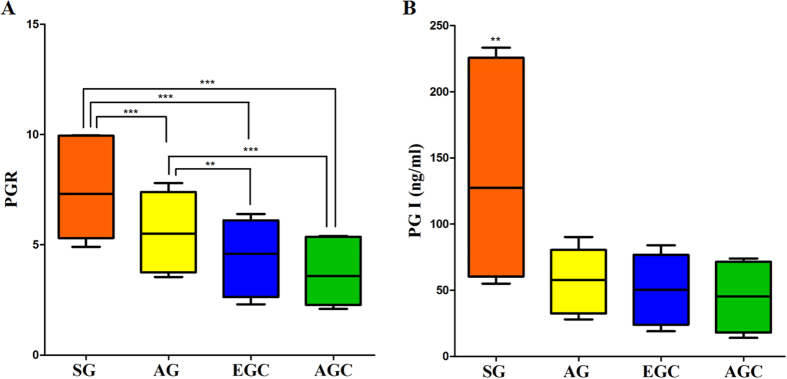
PG I and PGR in superficial gastritis group and atrophic gastritis group compared with gastric cancer. (**A**) PGR in atrophic gastritis group and superficial gastritis group were significantly higher than that of the gastric cancer group. B) Serum PG I concentration in superficial gastritis group was significantly lower than that of gastric cancer. (**P < 0.01, ***P < 0.001).

PG I and the PGR in AG group and GC group were significantly lower than that in the SG group. The difference was statistically significant (*P* < 0.05). In contrast, we did not find any difference in mean serum PG I concentrations and PGR between EGC and AGC. The value of PG I and PGR in different groups shown in Fig. 2A and B. In this study, P5 of PG I and PGR were 72.78 ng/ml and 4.15, respectively, using this cut off point to evaluate the sensitivity and specificity of serum PG in the diagnosis of GC. The sensitivity was 42.5% and the specificity was 95.6%, shown in Table 4.

**Figure 3:**
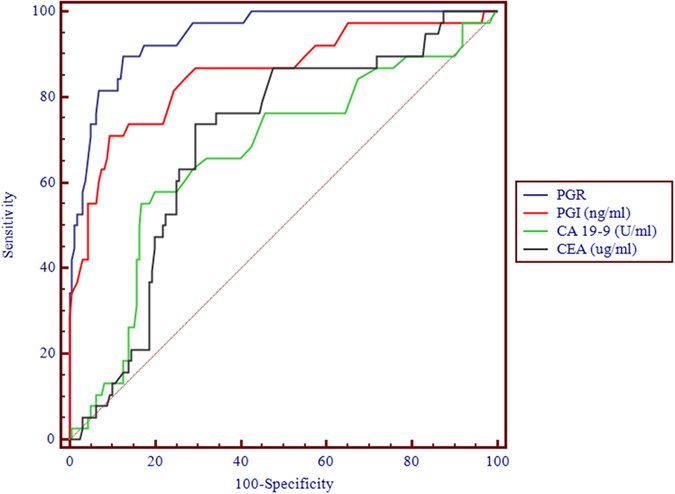
Receiver operating characteristic (ROC) curve analyses for the use of PG I/II ratios (PGR, blue line), pepsinogen I (PG I, red line), CA 19-9 (green line) and CEA (dark line) as serum markers for gastric cancer. The X axis shows 100 - specificity, while the Y axis displays sensitivity for PGR, PG I, CA 19-9 and CEA were 0.949, 0.858, 0.668 and 0.696, respectively. Serum PG test was better than serum CA 19-9 and CEA for use as a screening serum marker for gastric cancer.

Furthermore, GC as a case group, which was compared with the NC group which can draw receiver operating characteristic (ROC) curve of serum PG I and PGR (Fig. 3). Area under the curve (AUC) to evaluate the ROC curve which was close to one, the diagnosis effect was better. The AUC of serum PG I was 0.858 (95% CI: 0.801 to 0.903), AUC of PGR was 0.949 (95% CI: 0.905 to 0.974). The most suitable cut off point was a PG I concentration ≤64 ng/ml and PGR ≤4.5. Using this cut off point, the sensitivity and specificity of PG screening for Kazakh GC were 80.5% and 89.8%, respectively. These EGC cases were detected by serum PG screening for GC if the PG I concentration is set at less than 65 ng/ml and the PGR at less than 4.3 as the cut off point. Using this cut off point, the sensitivity and specificity of serum PG screening for Kazakh EGC were 80.2% and 89.5%, respectively. Meanwhile, of the 42 cases of AGC were detected by serum PG screening for GC if the PG I concentration is set at less than 63 ng/ml and the PGR at less than 4.2 as the cut off point. If we investigated the accuracy of PG screening using this cut off point for the sensitivity and specificity value would be 79.9 and 89.6% respectively.

### Comparison of diagnostic accuracies of serum PG test and CA 19-9 and CEA

Simultaneously, we analyzed serum levels of CA 19-9 and CEA in the same population, using ELISA. ROC analysis was performed to evaluate the diagnostic utility of PG test and CA 19-9 and CEA results, as these values were identified as independent biomarkers of GC. PG test performed better than CA 19-9 and CEA in distinguishing patients with GC from controls, with the AUC values for PGR, PG I, CA 19-9 and CEA were 0.949, 0.858, 0.668 and 0.696, respectively (Fig. 3). These results indicate that the PGR signature is a more accurate biomarker than CEA and CA19-9 for GC diagnosis.

## Discussion

Gastric cancer is one of the most common malignant tumors and the Kazakh population in Xinjiang has been reported to be one of the highest incidence of GC in the world. For a long time, the diagnosis of GC relies mainly on endoscopy and pathology. However, due to endoscopy and pathology operator had a higher requirement and poor patient compliance, failed to do well spread. Since most of EGC is at a curable stage, a serological screening with the appropriate biomarkers could contribute to decreasing the mortality of Kazakh GC. Therefore, to find a rapid, simple and reliable screening method for EGC is especially important.

Serum PG levels reflect the morphological and functional status of gastric mucosa. Both low serum PG I and a low PGR are recognized as serological markers of gastric atrophy. Human PGs have a diagnostic value for various gastroduodenal disorders, especially for peptic ulcer and atrophic gastritis, which have been widely discussed^11^,^12^. The PGR can provide even better information on the extent of chronic gastritis than gastric intubation^13^. With the progressive development of gastric diseases, time of gastric atrophy which main gastric glands gradually disappear and the loss of related functions, then primary cells were replaced by pyloric glandular cells. Finally, the secretion of PG I was significantly decreased, but the level of PG II was relatively unaffected, so the PGR would fall. Thus in the process of the development of gastric diseases, always showed that serum PG I increased first, and then decreased. PG II increased and maintained a high level. Furthermore, serum PG tests served as a useful marker for the prevalence of GC in a cross sectional setting. The results of the serum PG screening tests are comparable, and in some respects superior to those of traditional screening.

Compared with the conventional methods of ELISA and RIA, the method of flow fluorescence technology which enabled PG I, PG II in the same reaction unit parallel detection and also can effectively improve the clinical detection efficiency^14^,^15^. The linear range of validation shown that PG I and PG II had a good linearity in the range of linear statement on the kit. The experiments also evaluated the serum PG I, PG II sensitivity by flow fluorescence which both less than 1 ng/ml. The precision of PG I and PG II in serum were assessed by method of flow fluorescence, results shown that within batch CV < 7% and between batch CV < 9%. Method comparison with ELISA, shown that the two methods to detect PG I and PG II with good correlation. Due to the different methodology and the source of the test standards was different, although in experiment the results of flow fluorescence detection were generally slightly lower than the results of the ELISA, there was a systematic error between the two methods. However, linear range in two methods to detect PG I, PG II were closed to and had good correlation. In addition, the PG I and PG II quantitative detection kit of flow fluorescence technique had a good antigen-specific, recovery experiments shown a higher recovery rate and detection of serum common interfering substances with good anti-jamming capability. We found that the concentration of Hook effect occurs with respect to upper limit of PG I and PG II in normal population which had a larger gap. Therefore, it can effectively avoid the Hook effect.

In this study, the serum samples of 160 healthy people were detected. PG I and PGR in healthy population were higher than that in patients with AG and GC. Thus, it is estimated only reference range lower limit of healthy. Calculated using the non-parametric estimation method, P5 of PG I and PGR were 72.77 ng/ml and 4.16, respectively. P5 of PG I and PGR were closed to the literature report (PG I > 70 ng/ml, PGR >3)^16^,^17^. This experiment by flow fluorescence method also confirmed that the concentration of serum PGR, PG I and the degree of pathological changes were negatively correlated in AG and GC patients. After statistical analysis the difference was statistically significant, and the results of this study were consistent with the relevant literature reports^18^,^19^.

The significant reduction of PG I and PGR were valuable in screening GC. In 1998, Yashihara *et al*. implemented PG and gastroscopy in 10966 healthy residents, 90% belong to the early for detection of GC which far higher than conventional clinical early diagnosis of 56.9%^20^. In 1999, Kitahara *et al*. have examined 5113 people with PG plus endoscopic biopsy, with serum PG I < 70 ng/ml and PGR <3 were a critical value which the sensitivity of the diagnosis of GC was 84.6% and specificity was 73.5%^11^. Dinis-Ribeiro *et al*. conducted a systematic review of the diagnosis of GC in 42 articles about serum PG, the results showed that the optimal critical values of different populations were not consistent^21^. In our study, P5 of PG I and PGR were 72.78 ng/ml and 4.15. Using this cut off point, the sensitivity and specificity of PG screening for Kazakh GC were 42.5% and 95.6%, respectively. Further analysis of ROC curve, the AUC of serum PG I and PGR were 0.858 and 0.949, respectively. These results suggested that it has a high diagnostic value of GC. ROC curve had ability that can easily identify any boundary value of the disease and select the best diagnostic threshold. The results of this study shown that associated criterion of Kazakh GC were: PG I ≤ 64 ng/mL and PGR ≤ 4.5. Using this cut off point, the sensitivity and specificity of pepsinogen screening for Kazakh GC were 80.5% and 89.8%, respectively. More importantly, in this observational study, serum PG from patients with EGC were significantly higher than those of SG and AG. Moreover, the AUC of the PGR for GC diagnosis was 0.949, which was significantly higher than that of combined tumor markers. Thus, detection of serum PG by method of flow fluorescence in the diagnosis of Kazakh GC had good specificity, and it were significant for the screening in Kazakh GC. Especially for EGC, the serum PG screening for early detection of Kazakh GC is very important. It could contribute to decreasing the mortality of Kazakh GC. However, it can be used as the auxiliary diagnosis but not be used as a diagnostic test for GC.

In Japan, Finland, Norway and other countries, serological tests used in screening for GC and gastric precancerous lesions at high risk, and it also had good economic benefits and social benefits. The detection rate of GC was increased, and the mortality of GC was decreased. But throughout screening tool in these countries, on the basis of serum PG screening of high-risk groups followed by x-ray and upper gastrointestinal endoscopy. It can complement each other. Not only makes up the limitation and heterogeneity of the single detection of serum PG, but also to avoid unnecessary radiation exposure and pain caused by endoscopic examination.

In summary, these findings suggest that serum PG test can be used effectively in Kazakh GC in Xinjiang, China. In addition, not only the flow fluorescence assay can conduct simultaneous detection of PG I and PG II, but also give good performance. Meanwhile, the serum PG test has many advantages, including simple, inexpensive and its suitability for combination with other screening methods. Due to its reasonable sensitivity and specificity for early diagnosis in GC and significant advantage over the other tumor markers, serum PG test could be as a potential circulating predictor for Kazakh EGC diagnosis and screening.

## Materials and Methods

### Ethics Statement

Each participant provided written informed consent before enrollment in this study, and the protocols were approved by the institutional ethics committee at Yili Friendship Hospital in accordance with the ethical guidelines of the Helsinki Declaration. The methods were carried out in accordance with the approved guidelines.

### Study population

All participating was recruited from the Yili Friendship Hospital in Xinjiang, China. From November 2014 to January 2017, individuals who referred to gastrointestinal clinics for endoscopic examination, were invited to participate in our study. The exclusion criteria included history of chemotherapy or gastric surgery, history of helicobacter pylori eradication, history of anti-coagulant therapy and serious systemic diseases including diabetes, liver cirrhosis, and chronic renal failure.

### Gastroendoscopy

It was carried out at an endoscopy unit of the Yili Friendship Hospital with topical pharyngeal lidocaine anesthesia by skilled endoscopists (over 5 years’ experience), using standard gastroendoscopes (GIF-P30, GIF-XQ260; Olympus, Tokyo, Japan). If mucosal appearances were suspected of cancers during gastroendoscopy, endoscopic biopsies were taken from the areas. After biopsy materials had been examined in the pathology laboratory. Based on the pathological examination, subjects were classified into four groups including normal, SG, AG, EGC and AGC.

### Serum samples

Blood was taken from members of the patient and control groups after fasting for 12 hours. Sera were collected by centrifugation at 4,000 rpm for 5 min. Each serum sample was stored in two individual tubes and stored at −80 °C until analysis. Prior to the measurement of the serum PG I and PG II levels, serum samples were thawed in a refrigerator at 4 °C for 12 hours and then were brought to room temperature.

### Flow fluorescence detection

According to PG I/PG II flow type fluorescence quantitative reagent kit (Shanghai Tellgen Life Science Co., Ltd, China), detection of serum PG I and PG II levels in the Luminex200 multifunction flow type dot matrix analyzer (Luminex Co., Ltd, USA). PG I/PG II flow fluorescence quantitative assay kit.

### ELISA

According PG I/PG II ELISA kit (Biohit company, Finland) instructions, and the sample were added with full automatic enzyme immunoassay analyzer. According to the above methods, the levels of serum PG I and PG II were detected in patients with SG, AG and GC. CA19-9 was also measured using a commercially available ELISA assay. Personnel blinded to the diagnosis of patients performed all analytical measurements.

### Statistical analysis

Analysis of data was performed using SPSS 17.0 (Chicago, IL, USA). Using Kol-mogorov-Smimov test was analyzed for whether the data was normal distribution, for normally distributed measurement data between the two groups used independent sample t test. Variance analysis was used in multiple groups, and SNK-q test was used for pairwise comparison. Correlation analysis between the two methods used Person correlation analysis. Percentile method was determined the normal reference range. Skewed distribution of measurement data was showed as median and interquartile range [M (P25~P75)]. Kruskal-Wallis H test was used for comparison between multiple groups. *P* < 0.05 was considered statistically significant.

## Additional Information

**How to cite this article:** Juan Cai, W. *et al*. The Serum Pepsinogen Test as a Predictor of Kazakh Gastric Cancer. *Sci. Rep.* **7**, 43536; doi: 10.1038/srep43536 (2017).

**Publisher's note:** Springer Nature remains neutral with regard to jurisdictional claims in published maps and institutional affiliations.

## Figures and Tables

**Table 1:** Characteristics of 106 Patients with Biopsy-Confirmed Gastric Cancer.

Characteristics	Value
Sex	
Male	65 (61.3%)
Female	41 (38.7%)
Age (years)	53.3 ± 13.8
Location	
Antrum	45 (42.5%)
Body	47 (44.3%)
Antrum and body	10 (9.4%)
Cardia	4 (3.8%)
Subtypes	
EGC	64 (60.4%)
AGC	42 (39.6%)
Lauren classification	
Intestinal	52 (49.0%)
Diffuse	50 (47.2%)
Mixed	4 (3.8%)
Symptoms	
asymptomatic	72 (67.9%)
symptomatic	34(32.1%)
Smoking	
Never	38 (35.8%)
Current	30 (28.4%)
Ex-smoker	38 (35.8%)
Alcohol	
None	51 (48.1%)
Social	37 (34.9%)
Heavy	18 (17.0%)*

Data are presented as mean ± SD or number. EGC, early gastric cancer; AGC, advanced gastric cancer. *More than 200 g/wk.

**Table 2:** The average recovery rate of PG I and PG II which were detected by flow fluorescence method.

Project	number	Base C (ng/mL)	Added C (ng/ml)	Recovery C (ng/ml)	Recovery (%)	Average recovery(%)
PGI	1	4.39	80	80.1	94.6	95.4
	2	4.39	160	158.3	96.2	
PGII	3	2.62	28	28.3	91.7	94.9
	4	2.62	84	85.0	98.1	

Note: C, concentration.

**Table 3:** Data of interference experiment.

Project	Interferences	Interferent C (mg/ml)	Base C (ng/ml)	Interference samples C (ng/ml)	Recovery rate (%)	Explain
PGI	bilirubin	10	17.63	18.03	102.3	Hemolytic interference
			80.67	77.67	96.3	
	hemoglobin	10	24.07	23.77	98.8	Hemolytic interference
			79.61	71.40	89.7	
	triglyceride	200	19.67	18.88	96.0	Lipemia interference
			80.28	78.35	97.6	
PGII	bilirubin	10	5.77	6.07	105.2	Hemolytic interference
			35.33	34.77	98.4	
	hemoglobin	10	5.40	5.17	95.7	Hemolytic interference
			34.87	31.25	89.6	
	triglyceride	200	6.04	5.61	92.9	Lipemia interference
			38.34	37.50	97.8	

**Table 4:** Sensitivity and specificity of serum PG for diagnosis of gastric cancer.

Method	Confirmed by endoscopic biopsy			
	+	−		Total
Flow fluorescence	+	45	7	52
	−	61	153	214
Total		106	160	266
